# Feasibility and comparative analysis of *Dirofilaria immitis* microfilaria freezing and fixation for student instruction and assessment of clinical parasitology skills

**DOI:** 10.1186/s12917-020-2248-3

**Published:** 2020-01-31

**Authors:** Sidney A. Long, Jaylyn Rhinehart, Jessica Shrake, Antoinette E. Marsh

**Affiliations:** 1 Department of Veterinary Preventive Medicine, College of Veterinary Medicine, The Ohio State University, 1920 Coffey Road, Columbus, OH 43210 USA; 2 Department of Veterinary Clinical Sciences, College of Veterinary Medicine, The Ohio State University, 601 Vernon Tharp Drive, Columbus, OH 43210, 531 USA

**Keywords:** Heartworm, *Dirofilaria immitis*, Teaching, Mf, Cryopreservation, Diagnostics

## Abstract

**Background:**

Detection of *D. immitis* microfilaria (mf) is an important diagnostic skill in veterinary medicine and is critical to Day 1 veterinarians and technicians. Finding a supply of blood containing mf to teach the technique and formalin’s adverse environmental effects used in the diagnostic microscopic tests present a challenge.

**Results:**

This study evaluated the use of cryopreserved and recently drawn mf-infected blood along with two fixative reagents, acetic acid or formalin for mf detection. The specific aims included determining if veterinary students could 1) detect cryopreserved mf added to fresh blood using routine diagnostic testing and 2) detect morphological differences in the mf. The 236 students were kept blind from the sample status. The ability of the students to identify mf and the mf morphology were compared for the samples and fixatives evaluated. The results demonstrate using a combination of cryopreservation and acetic acid for teaching microfilaria diagnostic techniques is fleasible; however, the quality of the mf morphology is less than optimal when compared to freshly acquired mf containing blood. Compared to reference values, the mf demonstrated a decrease in size with each additional variable evaluated.

**Conclusion:**

A majority (98.3%) of the 236 students correctly identified the presence of mf. Teaching laboratories could utilize cryopreserved mf-spiked donor blood in lieu of freshly collected mf-containing blood from a naturally or experimentally infected dog. Substitution of less hazardous chemicals for the fixative can be used. Finally, the change in size measurements provides a mechanism to ensure students can correctly measure mf as students are required to do verifiable measurements and cannot copy reference values from a text book since the cryopreservation and fixation methods cause the mf to measure smaller than textbook reference values.

## Background

*Dirofilaria immitis*, the causative agent in heartworm disease, is spread worldwide through infected mosquitos. The main reservoir for *D. immitis* is the domestic dog, but human, felid, and wildlife infections are documented [1]. In 2018, the Companion Animal Parasite Council compiled 143,492 cases of heartworm disease in dogs. This is a significant increase in heartworm positive cases from just 5 years prior when in 2013, 77,557 cases of heartworm disease in dogs were reported (CAPC Database). Both climate change and increases in the interstate and international transport of dogs have increased the prevalence of heartworm disease around the world [1, 2]. Microfilaremic dogs can serve as a reservoir for parasite transmission. Finally, cases are costly to treat and treatments can be associated with deleterious side effects. All of these factors increase the need for veterinarians to be able to correctly diagnose *D. immitis* infection [1]. Diagnosis of canine dirofilariasis is critical to public health as humans can be an incidental host of *D. immitis* and suffer from clinical signs related to infection [3].

*Dirofilaria immitis* diagnostics usually includes an antigen test [1]. However false negatives due to antibody-antigen blocking can occur [4]. The importance of knowing how to test for microfilariae (mf) in blood samples is critical for veterinarians and technicians. Two main diagnostic techiques exist for mf detection: the Modified Knott’s and carbonate filter tests [5]. The competency of veterinarians and technicians to perform these tests and recognize mf is reliant on the teaching of the techniques during their clinical laboratory skills education. Often a supply of *D. immitis* mf infected blood can be difficult to obtain when scheduled to teach these diagnostic skills. To address the need for a consistent source of *D. immitis* mf, cryopreservation of the mf was evaluated. Successful cryopreservation of *D. immits* mf would allow instructors to have a stock of mf in the laboratory freezer for teaching purposes, without having to obtain recently drawn “fresh” mf containing blood in sufficient volume when students want to practice diagnostic techniques.

Not only is banking the source for *D. immitis* mf important, but the use of “environmentally friendly” fixatives creates a need for evaluating modifications to diagnostic teaching and the materials used in these assays. Both the Modified Knott’s and the carbonate filter procedures use a 2% formalin fixative [5]. Formalin is labeled as hazardous waste in most laboratories due to the toxicity to humans and the environmental implications [6]. Initial research on comparing 2% formalin versus glacial acetic acid in a clinical setting when performing the Modified Knott’s test suggests that 2% acetic acid can be substituded in place of 2% formalin fixation for easier disposal of the liquid waste and lower potential health risks [7]. Therefore, this study reports on the evaluation of mf cryopreservation techniques used in conjunction with environmentally friendly 2% acetic acid in a teaching laboratory used for training and assessment of veterinary students.

## Results

### Year 1 evaluating fleasibility of cryopreservation and spiking donor blood

**Fig. 1:**
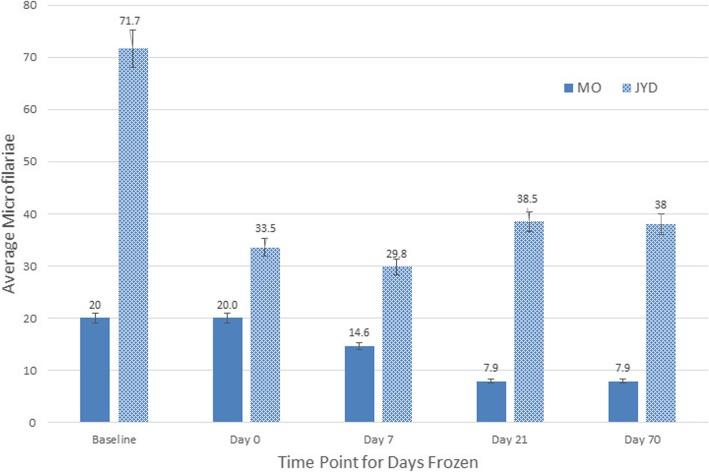
Average number of microfilariae recovered per study timepoint, baseline, Day 0, Day 7, Day 21 and Day 70 after cryopreservation using Year 1 protocol and the Modified Knott’s test. Counts were preformed on 10 random microscope fields using the 10x objective

The mf that were recovered on days 0, 7, 21, and 70 day remained above 897 mf per 50 ul of Modified Knott’s test (Table 1). The size of the mf varied and were smaller than reference values 280-320 μm × 6.1–7.2 μm (8) and 295-325 μm × 5.0–7.5 μm [5]. Both isolates experienced a decrease in head scoring the longer the mf were frozen. There was also a decrease in average mf concentration following cryopreservation and then the concentration remained relatively static thereafter (Fig. 1). Throughout the freezing time evaluation, the JYD isolate had a greater concentration of mf per ml as compared to the MO isolate.

**Table 1:** Modified Knott’s test recovery comparison of *D. immitis* microfilaria, MO and JYD isolates, using Year 1 cryopreservation procedure

Day	Isolate	Recovery	Measurements	Size	Score
0	MO	> 1000	215.0–255.0 × 2.5–5	237.0 × 3	2.4
7	MO	> 1000	257.5–312.5 × 5	288.0 × 5	2.1
21	MO	1189	255.0–300.0 × 5	276.8 × 5	2.2
70	MO	897	222.5–270.0 × 2.5–5	240.5 × 4.8	2.1
0	JYD	> 1000	257.5–292.5 × 5	274.0 × 5	3
7	JYD	> 1000	257.5–302.5 × 5	275.5 × 5	2
21	JYD	> 1000	250.0–292.5 × 5	271.8 × 5	2
70	JYD	> 1000	237.5–270.0 × 5	253.0 × 5	1.9

Day 0, no cryopreservation added, Day 7, 21, and 70 cryopreservation added followed by freezing at − 20 °C

MO and JYD isolates from experimentally infected dogs

Modified Knott’s slide with 50-uL of sediment, number of microfilaria detected

Range for the 10 microfilaria measured in μm

Average length and width of the microfilaria measured in μm

Average Head Score- 1-3 scale, 3 = clear and distinguished head; 2 = stained head but lighter than body; 1 = stained same color as body or indistinguishable

The average length and width of cryopreserved mf measured by 38 pairs of veterinary students was 260.5 μm by 7.7 μm. The average length and width of fresh mf measured by 21 pairs of veterinary students was 292.3 μm by 12.2 μm. All of the 118 veterinary students correctly identified mf in c-mf+. Two students commented that the mf in c-mf + were smaller than expected and seven students commented on the abundance of mf present.

During Year 1, a significant disadvantage occurred as the Glycerolyte 57 solution inadvertently caused cryopreservation of the blood cells along with the mf in the mf-infected whole blood. Later, these cryopreserved host cells did not lyse well when exposed to the 2% formalin. When the student performed the carbonate filter test, the filter trapped intact non-lysed blood cells caused difficulties in expressing the solution through the filter as compared to the nc-mf + samples. Many students also commented about the excess amount of erythrocytes present on the resulting slide-filter preparation.

### Year 2 evaluating the preformanace of semi-purified frozen microfilaria with either 2% acetic acid or 2% formalin

All of the 118 veterinary students correctly identified the presence of mf in their c-mf + sample using 2% acetic acid fixative. One pair of students reported a false negative when evaluating a c-mf + sample using the 2% formalin with the carbonate filter test. An instructor confirmed that the carbonate filter had dried out under the microscope. One pair of students reported a false negative result from a nc-mf + sample using the 2% acetic acid sample in the carbonate filter test. An instructor confirmed that the students did not mix the blood sample and pipetted the blood from the top of the sample. Microfilariae were found in the tube’s residual ~ 200 ul of blood sample using the Modified Knott’s test following the release of the coded laboratory exercise. Two pairs of students that were given mf- samples reported their samples as positive for mf using the carbonate filter test. One pair measured their purported “mf” to be 690 μm and the other pair measured their purported “mf” to be 84 μm.

The 2% acetic acid fixative caused the blood to turn significantly darker than with 2% formalin. The 2% formalin also created a darker staining of mf as compared to the 2% acetic acid fixation. The 2% acetic acid in combination with c-mf + caused a greater decrease in the average length of the mf compared to any other fixative/mf combination.

During the initial phase to semi-purify and cryopreserve the mf, the mixture of red blood cells and mf underwent a lysis step prior to mf cryopreservation to mitigate the problem of cryopreserved erythrocytes and blood cells experienced during Year 1. This modification improved the assay technique (less filter backpressure) and the quality of the slides (fewer background erythrocytes). An unanticipated observation from the Year 2 technique of cryopreservation when stored at − 80 °C included visualizing motile mf in a drop of the thawed sample. However, the time duration for mf to remain motile was not measured.

Table 2 shows the average length and width reported by the veterinary students and the experienced diagnosticians for each assay type. The length and width followed the same decreasing measurement value trend for both the diagnosticians and students in this order: nc-mf + in 2% formalin > nc-mf + in 2% acetic acid > c-mf + in 2% formalin > c-mf + in 2% acetic acid.

**Table 2:** Measurements in Year 2 using the Modified Knott’s test

Assay type	Diagnosticians (*n* = 2)		Students (*n* = 118)		n
	length	width	length	width	
Non-cryopreserved mf, 2% formalin	306.2	5.5	302.8	8.3	22
Non-cryopreserved mf, 2% acetic acid	298.3	5.8	297.3	8.3	21
Cryopreserved mf, 2% formalin	281.2	5.5	277.8	6.4	23
Cryopreserved mf, 2% acetic acid	283.0	5.4	256.7	6.3	28

n- total number of students evaluating the sample and fixative

Measurements in microns

Both experienced diagnosticians ranked a nc-mf + sample as the best overall morphology and slide quality. Overall, diagnostician A ranked the slides from best to worst in the following order: nc-mf + in 2% formalin (best), then c-mf + in 2% formalin, then nc-mf + in 2% acetic acid, and c-mf + in 2% acetic acid (worst). Diagnostician B ranked the slides from best to worst in the following order: nc-mf + in 2% acetic acid (best), then nc-mf + in 2% formalin, then c-mf + in 2% formalin, and c-mf + in 2% acetic acid (worst). Both diagnosticians made comments that the 2% acetic acid stained lightly whereas the 2% formalin stained more darkly, and that the nc-mf + samples created straighter mf as compared to the c-mf + samples.

### Assessment of clinical students during their hospital rotation

All eleven of the fourth year clinical students identified the c-mf + sample as mf+. Three of the eleven clinical students identified the mf- sample as mf+. The measurements of the purported “mf+” were 1500 μm × 5 μm, 110 μm × 2 μm, and 450 μm × 5 μm which indicates the students were not actually looking at mf as that particular sample contained none of the purported mf. There were 6 of the 11 students that attempted to identify the microfilaria with the following results: 3 identified *Acanthocheilonema reconditum*, 2 identified *D. immitis*, and 1 identified *Dirofilaria repens*. For true positive samples containing the cryopreserved mf spiked sample, the average mf measurement was 270.5 μm × 6.7 μm (*n* = 11).

## Discussion

The MO and JYD isolates used contained a high concentration of mf. Even with a low recovery of mf it is possible for the students to obtain mf for identification if the starting concentration of mf is high. Blood with a very low concentration of mf may not be an adequate candidate for cryopreservation. There is a drop in the number of mf recovered when the blood is cryopreserved compared to the freshly acquired non-frozen mf-infected blood. Once cryopreserved, the concentration of mf recovered appears to remain relatively stable over the time as evaluated in this study (Fig. 1).

In the teaching laboratory veterinary students being taught the diagnostic technique did not have difficulties identifying the presence or absence of mf in the samples regardless of cryopreservation, fixation, or technique in either Years 1 or 2. The cryopreservation procedure consistently caused a decrease in mf average length and width compared to non-cryopreserved mf. This is a concern for specifically teaching parasite identification based on size because the reduced size of the mf led the students to believe that the mf were not *D. immitis* but a different parasite. Five pairs of students in the laboratory course misidentified the mf and thought they were *A. reconditum* based on size. *Acanthocheilonema reconditum* length is 250–288 μm and width 4.5–5.5 μm [5]. Moreover, it must be considered that the students are inexperienced with viewing the distinctive morphology of the *D. immitis* mf, and this was the first time the students performed these techniques in the teaching laboratory to view the mf microscopically. All five pairs of students that identified the *D. immitis* mf as *A. reconditum* had c-mf + and four of the pairs of students used 2% formalin while one pair of students used 2% acetic acid. These students reported 220–275 μm for their measurements. The students who reported false negatives experienced technical difficulties as one student pair allowed their filter (carbonate filter assay) to dry out during their prolonged slide reading (> 45 min), and the other student pair failed to properly mix the blood sample tube prior to preforming the assay. Thus, it is important for instructors to monitor the students preforming the assays to ensure they are compliant with standard operating procedures.

**Fig. 2:**
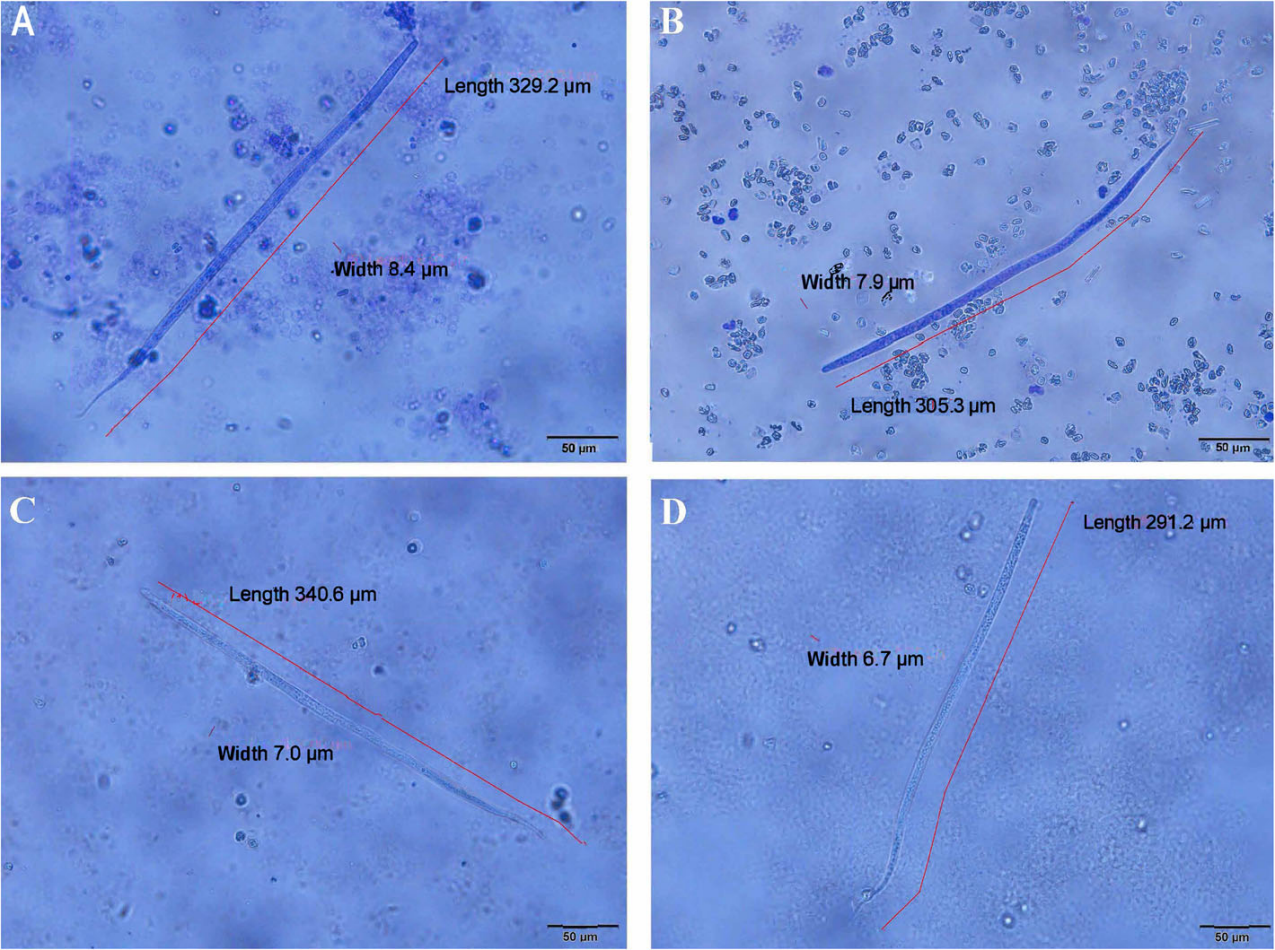
Modified Knott’s test slide preparations using variable testing conditions in Year 2 with microfilariae (mf) length and width measurements in microns and variable background erythrocytes and host cells. **a**. Non-cryopreserved mf with 2% formalin fixative. **b**. Cryopreserved mf with 2% formalin fixative. **c**. Non-cryopreserved mf with 2% acetic acid. **d**. cryopreserved mf with 2% acetic acid

Cryopreserved mf do not straighten like non-cryopreserved mf when exposed to fixative. This can lead to difficulties in measuring the mf and may be responsible for small discrepancies between student measurements. However, measurements of the mf using digital photomicrographs with software to include measuring the mf curvature produced similar measurements as the students (Fig. 2).

The mf recovered in Year 1 were given a head score and cuticle score to compare cryopreserved mf that had been frozen for different time points. The head and cuticle scoring are both subjective data. The identification of mf relies on measurements and the tail and head morphology to distinguish species. The mf morphology quality score and measured average length decreased following cryopreservation. However, the 124 day post-freezing samples were sufficient for teaching the technique and allowing visualization of the mf recovered despite issues with the mf measurements and morphology.

A study evaluating the use of frozen-thawed human tissues for suture technique education determined that cryopreserved human tissues can serve as a teaching alternative that provide similar characteristics to original, natural tissues [8]. Practical techniques for mf cryopreservation were considered for use in a teaching laboratory similar to how human cryopreservation studies have addressed the practical use of tissue [8, 9]. The mf were stored at − 20 °C and at − 80 °C with no detectable changes in the mf. Most laboratories will have a − 20 °C freezer. Not all teaching laboratories will have access to a − 80 °C freezer or liquid nitrogen, and cryopreservation needs to be applicable to general teaching facilities.

Easy disposal of waste after performing the assays led to the inclusion of 2% acetic acid fixative in place of 2% formalin for easier disposal, safer handling, and as an environmentally friendly option. Water was initially evaluated during the pilot freezing trial, but because of the variation in measurements relative to reference values it was not pursued for the student evaluation trials. Nonetheless, the 2% acetic acid did cause noticeable changes to the blood coloration, mf-staining, and size as compared to the 2% formalin. These issues should be addressed with the students if the students, when in practice, plan to use 2% formalin solution as specified in reference manuals for the technique protocols. Therefore, further optimization of the acetic acid percentage used with the c-mf + could result in better staining, but it may not completely resolve the disparent size relative to associated reference values when using 2% formalin with freshly drawn blood containing mf.

Clinical students in their fourth year rotation could confidently detect mf from a positive sample. A few students detected artifact and identified the structures as mf despite having a negative sample. All of the artifacts detected as “microfilaria” had measurements well out of the range for normal mf, supporting that students are in need of more practice to become more comfortable reading slides. Many veterinary schools have incorporated clinical skills laboratories into their program and cryopreservation to bank a source of mf could allow students more opportunities to practice these techniques. Ordering fresh mf infected blood throughout the year for a clinical skills laboratory is impractical, costly, and *D. immitis* infected research dogs are limited in the frequency they can be bled. Cryopreservation allows for less frequent bleeding and a more consistent source of mf for teaching.

## Conclusions

The majority of the 236 laboratory course students from Year 1 and Year 2 were able to correctly detect mf from a “positive sample” either their prepared slide or viewing another pair’s slide. All 28 students with the 2% acetic acid and c-mf + combination correctly identified the presence of mf. Of the 236 laboratory course students that participated 234 (99.2%) were able to identify mf from a c-mf + sample regardless of fixative or technique. In some cases, they relied on another student pair’s prepared slide. Banking the source for mf and the practical use of 2% acetic acid could improve the teaching of diagnostics for heartworm disease. Eleven students were evaluated during their clinical hospital rotation. All the students could perform the Modified Knott’s assay but 3 out of 11 still reported false positive results. This method is suitable as a stand-alone for practicing the diagnostic technique but the teaching laboratory should include a displayed slide of *D. immitis* mf representing the correct size and good staining to display appropriate morphology for students to compare for learning and practicing parasite identification. In a clinical skills laboratory, the use of c-mf + with 2% acetic acid fixative provides a reasonable alternative for teaching purposes and evaluating students’ diagnostic skills.

## Methods

### Year 1 evaluating fleasibility of cryopreservation and spiking donor blood

In Year 1, only the cryopreservation of *D. immitis* was evaluated. Two isolates of infected blood were obtained from the NIH/NIAID Filariasis Research Reagent Resource Center, JYD and MO strains, collected into heparized tubes and shipped overnight. One aliquot of fresh blood was removed and designated as a baseline and immediately evaluated using the Modified Knott’s test, [10] modified to use water to substitute for the 2% formalin. Photomicrographs were taken and the Modified Knott’s’ preparation was permanently mounted onto a slide. The remaining heparinized whole blood was mixed 1:1 with Glycerolyte 57 solution (Baxter, USA), divided into 1 ml aliquots and frozen at − 20 °C. One sample was then immediately thawed (Day 0) while the others remained frozen for later diagnostic test processing on days 7, 21, 70, and 124 post-freezing.

On days 7, 21, and 70 two samples of each isolate were removed from the freezer to perform a Modified Knott’s, again using water instead of the 2% formalin. Total volume of pellets were measured for recovery purposes. New methylene blue (Ricca Chemical Company, USA) was used to stain the microfilaria for all of the Modified Knott’s and carbonate filter tests performed in this study. The detected mf quantity and morphology were recorded for each time point and compared to baseline. Quantity data was collected by evaluating the total number of mf per slide and the average number of mf per 10x objective field. The average number of mf per field was found by taking 10 random views of the slide and averaging the number of mf seen. Morphology of ten individual mf was evaluated using a head scoring of 1–3, cuticle scoring of 1–3, and the measurements of the mf compared to reference values [5, 8]. The head scoring parameters were 3-clear and distinguished; 2-stained, but stained lighter than the body; 1-indistinguished, head and body same coloration. The cuticle scoring parameters were 3-crisp and defined edges of the body; 2-darkly stained; 1-unclear edge/no edge or damaged. Photomicrographs were taken at the different time points for comparison.

The day 124 post-freezing samples were used in the teaching laboratory to run a blind trial on 118 veterinary students. Instructors were also blinded. Pairs of students received a randomly assigned unknown microfilaria-status patient sample, coded by the investigator (SL), in a 3 ml ethylenediaminetetraacetic acid disodium salt dehydrate (EDTA) tube. The unknown patient samples consisted of one of the following: non-cryopreserved mf infected blood (nc-mf+), freshly acquired dog donor blood cells spiked with cryopreserved-infected blood (c-mf+), or non-infected normal dog donor blood cells (mf-). Acquisition of the normal dog donor blood cells came from a donor dog in the Ohio State Veterinary Medical Center Blood Bank program. For the dog donor blood, the plasma was removed and remaining blood cells were preserved using Adsol red cell preservative solution (Fenwal, Inc., USA) in the blood bank bag and kept at 4 °C.

The samples and required testing assays were coded to approximately distribute a variety of the samples and assays in each of the six laboratory sessions. The student pairs randomly drew their assigned patient and method, either a Modified Knott’s or carbonate filter assay with the standard 2% formalin fixation component. The student pairs reported if their sample contained detectable mf and if so, measured one or more mf on their slide. If their sample contained no mf they were required to view a positive slide from a different student pair and make written observations about the slides. Students submitted their written results worksheet for this exercise.

### Year 2 evaluating the preformanace of semi-purified frozen microfilaria with either 2% acetic acid to 2% formalin

In Year 2, further refinement of the study parameters and a new set of 118 students were evaluated. Changes included purification of mf prior to the addition of cryoprotective medium and the use of 2% acetic acid as a fixative in the techniques. Again the JYD and MO strains of *D. immitis* were used. For mf purification, heparinized whole blood containing mf was centrifuged for 10 min at 400 G at 25 °C. The separated layers of plasma, buffy coat, and red blood cells (RBC) were removed and a 10 ul aliquot was examined to check for mf. As the RBC layer contained sufficient living mf, further processing of this layer occurred. The 1 part of the RBC-mf layer was mixed with 9 parts of water and gently inverted for 1 min to lyse the host cells. Physiological isomolarity was returned using 9% sodium chloride solution. The mixture underwent centrifugation for 10 min at 400 G to pellet the mf, the resulting pellet was resuspended in an equal volume of Glyerolyte 57, 200 ul aliquoted into individual cryovials, rapidly frozen on dry ice, then stored at − 20 °C until the day before class.

One day prior to the laboratory teaching session, the mf samples were rapidly thawed at 38 °C and added to pre-labeled 3 ml EDTA tubes containing 1 to 2 mls of healthy donor dog blood cells. Similar to Year 1, the 118 blinded veterinary students were randomly assigned a diagnostic technique (Modified Knott’s or carbonate filter), a sample (nc-mf+, c-mf+, or mf-), and fixative (2% acetic acid or 2% formalin) to analyze. Instructors and students were kept blinded from the sample identity and fixative. Fixative bottles were labeled A or B. Students were paired with another student, and each pair randomly drew a patient sample associated with an assigned technique and fixative. Students were required to report if mf were present, if so, measure at least one mf in their slide, observe another student pair’s prepared slide, and make comments about the slides. We did not specifically require students to identify the microfilaria to genus and species. Based on the drawn sample’s patient name, students’ with c-mf + samples using the 2% acetic acid fixative were sent to look at a c-mf + sample processed with 2% formalin fixative and vice versa, so all students compared the two different fixatives relative to their microfilaria sample.

In Year 2 the samples were not evaluated on days 0, 7, 21, and 70 because the feasibility of storing cryopreserved samples was determined in Year 1.

A blind trial was conducted with two experienced laboratory diagnosticians A and B. The Modified Knott’s procedure was used to prepare four slides which consisted of the following: c-mf + in 2% acetic acid fixative, nc-mf + in 2% acetic acid fixative, c-mf + in 2% formalin fixative, and nc-mf + in 2% formalin fixative. The experienced diagnosticians were asked to rank the quality of the four slides based on the morphology of and the ability to detect mf. The best quality was ranked as 1 and the poorest overall as 4. The diagnosticians also measured two mf per slide. Photomicrographs were taken using a Olympus BX41 equipped with a DP74 camera (Olympus America Inc., USA) connected to a Dell Optiplex 7050 (Dell Technologies, USA) with cellSens Standard 1.18 software package (Olympus America Inc) for image acquisition and measurement tools.

### Assessment of clinical students during their hospital rotation

A blind trial was conducted on fourth year veterinary students (*n* = 11) during their clinical rotations. Two different student rotation groups were evaluated. This required the thawing and adding of cryopreserved microfilaria to healthy donor dog blood cells in EDTA tubes either 1 day or 14 days prior to the student assessments. During this 2 week period the spiked blood was kept at 4 °C. Each student was provided with 2% acetic acid fixative and two “unknown labelled blood samples” with one being c-mf + (spiked) and the other mf- (normal dog donor blood cells). The students were instructed to independently perform a Modified Knott’s on the blood samples. Students were required to report on a submission form their results if mf were present, and if so, measure and attempt to identify at least one mf on their slide.

#### Ethics approval and consent

The Ohio State University Office of Responsible Research Practices Institutional Review Board reviewed and exempted protocol 2019E0658 under 45 CFR 46 (USA). The research was conducted in a commonly accepted educational setting, involving normal educational practices and did not adversely impact students’ opportunity to learn the required educational content. The research compared reagents used for an instructional technique and was therefore exempted from human subject research under 45 CFR 46.

## Data Availability

Companion Animal Parasite Council Parasite Prevelance Maps for Heartworm are publicly available at https://capcvet.org/maps/#2018/all/heartworm-canine/dog/united-states/. The datasets used and analyzed during the current study are available from the corresponding author on reasonable request.

## Abbreviations

mf: Microfilaria
nc-mf +: non-cryopreserved mf infected blood.
c-mf +: freshly acquired donor blood cells spiked with cryopreserved-infected blood.
mf-: non-infected normal dog donor red blood cells (mf-).
mf +: microfilaria positive.
EDTA: ethylenediaminetetraacetic acid disodium salt dehydrate
